# Developing a youth sustainability healthy active living intervention for chronic disease prevention and environmental sustainability through community engagement and codesign: protocol for a multimethod community-based participatory research study in Canada and Jamaica

**DOI:** 10.1136/bmjopen-2026-119261

**Published:** 2026-10-07

**Authors:** Rosain Stennett, Sujane Kandasamy, Diana Sherifali, Russell J de Souza, Dipika Desai, Matthew YW Kwan, Joanne A Smith, Thera Edwards, Georgiana Gordon-Strachan, Shrikant I Bangdiwala, Gita Wahi, Jeffrey Brook, Myles Sergeant, Alisha Krishanthan, Sneha Desai, Simon G Anderson, Sonia S Anand

**Affiliations:** 1 Mary Heersink School of Global Health and Social Medicine, Department of Medicine, Faculty of Health Sciences, McMaster University, Hamilton, Ontario, Canada; 2 The Starfish Environmental Society, Hamilton, Ontario, Canada; 3 School of Nursing, Faculty of Health Sciences, McMaster University, Hamilton, Ontario, Canada; 4 Department of Health Research Methods, Evidence, and Impact (HEI), Faculty of Health Sciences, McMaster University, Hamilton, Ontario, Canada; 5 Population Health Research Institute/Hamilton Health Sciences, Hamilton, Ontario, Canada; 6 Department of Child and Youth Studies, Brock University, St Catharines, Ontario, Canada; 7 Department of Community Health Sciences, University of Calgary, Calgary, Alberta, Canada; 8 Epidemiology Research Unit, Caribbean Institute for Health Research, The University of the West Indies, Mona, Kingston 7, Jamaica; 9 Department of Geography and Geology, The University of the West Indies, Mona, Kingston 7, Jamaica; 10 Tropical Metabolism Research Unit, Caribbean Institute for Health Research, The University of the West Indies, Mona, Kingston 7, Jamaica; 11 Department of Pediatrics, Faculty of Health Sciences, McMaster University, Hamilton, Ontario, Canada; 12 Dalla Lana School of Public Health, University of Toronto, Toronto, Ontario, Canada; 13 Family Medicine, Faculty of Health Sciences, McMaster University, Hamilton, Ontario, Canada; 14 Canadian Coalition for Green Health Care, Hamilton, Ontario, Canada; 15 Health Sciences Program, Western University, London, Ontario, Canada; 16 Arts & Science Program, McMaster University, Hamilton, Ontario, Canada; 17 George Alleyne Chronic Disease Research Centre, Caribbean Institute for Health Research, The University of the West Indies, Bridgetown, Barbados; 18 Glasgow-Caribbean Centre for Development Research, The University of the West Indies, Mona, Kingston 7, Jamaica

**Keywords:** Community-Based Participatory Research, QUALITATIVE RESEARCH, Young Adult, Community Participation

## Abstract

**Abstract:**

**Background:**

Most non-communicable diseases (NCDs) develop through multiple complex pathways that involve non-modifiable and modifiable risk factors. Prevention efforts optimally involve a life course approach that promotes healthy eating and physical activity because behaviours adopted early in adolescence and young adulthood are likely to be sustained and can prevent NCDs in later life. This research protocol describes a community-based co-designed study taking place in Canada and Jamaica that positions youth aged 15–25 as agents-of-change in their communities to increase knowledge and develop tools to adopt and promote sustainable practices and healthy active living.

**Methods:**

The SCORE! Youth Sustainability Healthy Active Living (SCORE! Youth-SHAL)—Community Engagement and Co-design is a multimethod community-based participatory research (CBPR) study to be conducted in two sequential stages. Stage 1 involves community contextual mapping, a maximum of two community engagement workshops along with key informant interviews with both youth and stakeholders. Stage 2 includes a co-design workshop with local youth to develop customised healthy active living activities. Participants will be recruited using convenience and purposive sampling. Workshops and interviews will be audio-recorded and transcribed. Data will be collected to understand the SHAL intervention’s feasibility, fidelity and contextual fit across diverse settings. Content and thematic analysis will occur concurrently and iteratively with data collection.

**Ethics and dissemination:**

This study has approval from Hamilton Integrated Research Ethics Board in Health Sciences, Brock University Research Ethics Board, The University of the West Indies—Mona Campus Research Ethics Committee, and Ministry of Health and Wellness, Jamaica—Advisory Panel on Ethics and Medico-Legal Affairs. Findings assist in finalising a SHAL intervention for youth in equity-deserving populations. We will also publish the study results in an academic journal and present at conferences, webinars and workshops.

**STRENGTHS AND LIMITATIONS OF THIS STUDY**

- Youth-centred co-design: youth are engaged throughout the research process as advisory committee members and research partners, strengthening the relevance, acceptability and sustainability of intervention development.
- Community-based participatory approach: collaboration with youth and community stakeholders ensures interventions are culturally relevant, context-specific and responsive to local priorities across diverse settings.
- Limited generalisability: findings may not be transferable to all youth populations because participating neighbourhoods were purposively selected and represent specific demographic and socioeconomic contexts in Canada and Jamaica.

## Introduction

 Non-communicable diseases (NCDs) account for 74% of deaths worldwide.^1^ They impose an enormous burden on individuals and families, strain healthcare systems, impede economic growth and are a leading cause of premature mortality. While traditionally observed in older adulthood, the onset of NCDs is increasingly prevalent among younger populations,^2^ with adolescence and young adulthood identified as a critical period for establishing individual behaviours and practices that impact lifelong health trajectories and broader community well-being.^3^ Concurrently, climate change and environmental degradation pose an existential threat to human well-being, with unsustainable food systems and sedentary lifestyles contributing significantly to both ecological harm and the burden of disease.^4–8^ This confluence of challenges necessitates a paradigm shift from siloed interventions to integrated approaches that synergistically address human behaviour and planetary health.^9 10^

NCDs typically develop through complex pathways involving non-modifiable (e.g., age, family history, genetics) and modifiable risk factors (e.g., dietary intake, physical activity). Risk factors for cardiovascular diseases, type 2 diabetes mellitus (T2DM) and certain cancers include obesity driven by physical inactivity and poor diet quality,^1^ further enhanced by high consumption of ultraprocessed foods (UPFs).^11 12^ Physical inactivity and poor diet quality are exacerbated by climate change.^13–18^ Extreme weather events and deteriorating air quality increasingly disrupt opportunities for outdoor recreation. At the same time, climate change threatens food security by degrading diet quality through agricultural disruption, reducing the nutrient content of staple crops and decreasing the availability and affordability of healthy foods such as fruits and vegetables. Optimal NCD prevention strategies should adopt a holistic life-course approach that accounts for climate-related barriers to healthy eating and physical activity, recognising that establishing resilient health behaviours early in childhood can substantially reduce NCD risk across the lifespan.

Historically, public health strategies have focused on individual behaviour risk factors and, to some extent, understanding and appreciating the role of genetics in NCD risk, but generally neglect to adopt a synergistic view of broader environmental and societal determinants.^9 19^ For example, greenhouse gas emissions impact food systems, affecting agricultural yields, food availability and prices. This drives a shift towards purchase and consumption of cheaper UPFs, which contribute to NCDs.^13 20^ UPFs possess larger carbon footprints than locally sourced whole foods. These environmental pressures interact with broader societal determinants, including limited access to affordable, nutritious foods and inadequate urban planning that restricts green space and opportunities for physical activity,^21^ alongside gaps in nutrition literacy and self-efficacy.^22^

Efforts to mitigate NCDs are most cost-effective when initiated early in life, targeting the formative years (early childhood through young adulthood).^23 24^ It is proposed that every US$1 of public funds invested in adolescent-focused NCD prevention could generate US$5 in increased human capital.^25^ Comprehensive intervention approaches to improve diet and activity levels among children and youth are most successful when they are contextualised to individuals’ culture and environment/community, have low barriers to adoption and leverage existing community structures.^10 26^ These efforts should emphasise sustainable diets and integrate nutrition education, physical activity, outdoor play and mental well-being. Including stakeholders at all stages of development and implementation is crucial to ensure that interventions are tailored to specific community needs and are sustainable.

This paper presents a protocol for a participatory framework developed through community engagement and youth co-design. This will inform the creation of an intervention aimed at increasing knowledge and providing tools to support environmental stewardship and healthy active living (e.g, production and consumption of local fruits and vegetables, reduced UPF consumption).

### Study aim

The aim of the SCORE! Youth SHAL (phase 1) study, is to co-design an intervention program with youth aged 15–25 years and community stakeholders. This study seeks to increase knowledge and tools for action that increase opportunities for sustainable practices (i.e., environmental stewardship) and healthy active living (i.e, production and consumption of local fruits and vegetables; and decreasing UPF consumption).

### Primary outcome:

The SCORE! Youth SHAL intervention.

## Methods

### Study design

We will employ a multimethod, community-based participatory research (CBPR) approach. CBPR is a collaborative research framework that fosters equitable partnerships between researchers and community members across all stages of the research process.^27–29^ This design facilitates the development of a formative process fostering co-learning and empowerment and leveraging the unique strengths each partner brings.^27^ Partners (youth and other relevant actors) contribute their expertise and share responsibilities to enhance understanding of Sustainability Healthy Active Living (SHAL) and integrate the knowledge gained with action to improve the health and well-being of the community and its members. This model ensures that community partners and researchers share expertise and make decisions together.^28 29^ Qualitative (interviews and workshops) and quantitative (surveys) data are performed in two sequential stages: (1) community engagement and (2) co-design (figure 1).

**Figure 1:**
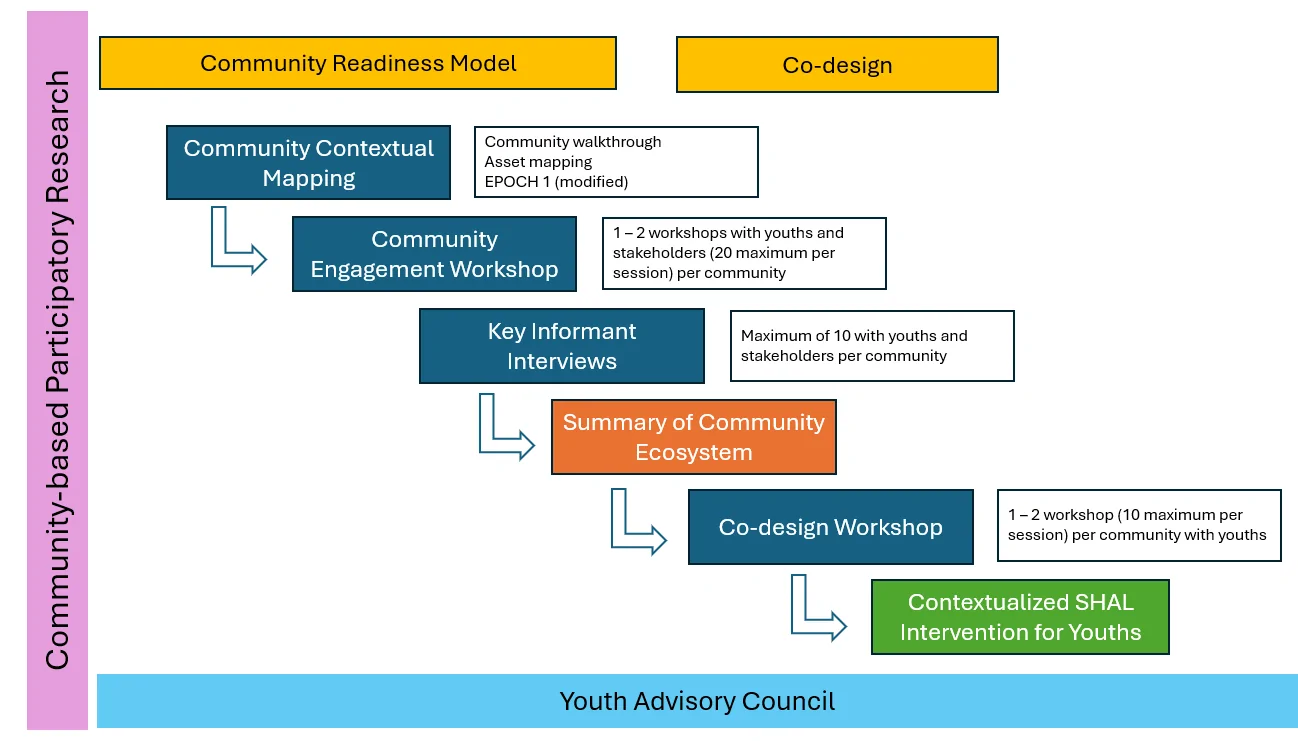
SCORE! Youth SHAL study flow. EPOCH 1 (modified): Environmental Profile of a Community’s Health; SCORE! Youth-SHAL: SCORE! Youth Sustainability Healthy Active Living.

### Setting

The study will take place in 11 communities/neighbourhoods across two countries:

- Hamilton, Ontario, Canada—two wards (treated as two neighbourhoods for this study).
- Calgary, Alberta, Canada—six neighbourhoods.
- Saint Andrew, Jamaica—three neighbourhoods.

The selected neighbourhoods in Hamilton and Calgary are urban neighbourhoods within cities where new immigrants and refugees settle when first arriving to Canada. Neighbourhoods in Jamaica are peripheral to the capital city of Kingston and were selected for their similarity in agriculture, schools, churches and community clubs (youth and elderly). Jamaica’s population is predominantly of African descent, with the largest ethnic minorities being of East Indian and Chinese heritage.

### Study timeline

The study required ethical approval by four research ethics boards, representing three academic institutions. The University of the West Indies (UWI) required approval from the UWI and the Ministry of Health and Wellness, Jamaica. Brock University required approval from Brock University Research Ethics Board, and McMaster University required approval from the Hamilton Integrated Research Ethics Board (HiREB). McMaster University obtained approval in November 2024, and this site began recruitment in December 2024. Data collection across all sites is expected to end in August 2026.

### Patient/public involvement

This study takes an inclusive approach by including youth as members of the research operations team to ensure that youth voices are heard and considered in the study decisions and planned activities. The study also seeks to establish a youth advisory committee to guide research activities. The committee is open to individuals aged 15–25 years who reside in one of the neighbourhoods or provinces involved in the study. Committee members serve a 1 to 5-year term (i.e., up to the full course of the study) and reflect the diversity of the target population with respect to age, gender, ethnicity, level of ability, sociodemographic factors and interests. Committee members will provide guidance on developing contextually relevant materials and study procedures to engage and involve youth. Members will also participate in skill-building activities and community-based initiatives to strengthen their capacity to serve as effective youth advocates.

### Stage 1—Community engagement

Community engagement activities are guided by principles of the Community Readiness Model, where the research team seeks to understand the ecosystem (e.g., services, environment, barriers and facilitators to SHAL) of each SCORE! Youth SHAL community through (1) community contextual mapping; (2) facilitated community engagement workshop(s); and (3) key informant interviews. Specifically, this stage aims to: (1) understand the community-level contextual factors (i.e, assets) that impact SHAL; (2) identify strengths, levers, challenges and barriers the communities face regarding SHAL; and (3) work with all relevant actors to identify levers and brainstorm approaches to the implementation of the SCORE! Youth SHAL intervention.

#### Community contextual mapping

Community contextual mapping seeks to understand the community and identify resources that are available for youth in the community. These efforts focus on conducting neighbourhood walks with youth and stakeholders; desk and document review (internet) of SHAL activities available at the community level; informal interviews with representatives of institutions/organisations that offer services to youth; and completion of the Contextual Factors Assessment Community Questionnaire—Environmental Profile of a Community Health (EPOCH) 1 (modified to local context).^30–33^

#### Community engagement workshop and key informant interview

##### Population

Within each community, we will invite a diverse group of participants to take part in a community engagement workshop or a key informant interview. These include youth aged 15–25 years, youth leaders who are actively involved in community activities (such as service clubs, societies or faith-based groups), and community members, including parents and other residents of the participating neighbourhoods. We will also engage community partners who work with youth in their professional roles—such as educators, social workers and members of the public health team—as well as organisational leaders who oversee youth-focused programs or services, such as school administrators or faith leaders. In addition, policy actors from relevant areas of governance (e.g., local members of parliament, ministries of education or health or public health officials) will be invited to contribute their perspectives.

##### Eligibility criteria

Any person residing or working within the selected neighbourhoods and belonging to one of the groups noted above who is willing to take part in a workshop and/or key informant interview, and able to provide informed consent or assent, as applicable, is eligible. Each meeting includes a mix of participants from across these groups.

##### Sampling strategies and framework

The study uses multiple sampling strategies to reach the desired population for each research activity. For both community engagement and key informant interviews, identification of participants will be driven by convenience (availability and interest in the study) and purposive sampling (key informants who meet inclusion criteria).^34^ Selecting people as key informants to be interviewed will use additional sampling techniques, including stratified (blending of community members and youth) and snowball sampling (asking current participants to refer others).^34^ The following sample size and approach allow for diversity of stakeholder perspectives and are deemed sufficient to achieve information power:

1. Community engagement workshop(s)—1–2 workshops per community, with approximately 20 participants at each workshop. A second workshop might be required if there is insufficient diversity or engagement of participants from the first workshop.
2. Key informant interviews—sample size of approximately 10 key informants per community/neighbourhood (based on community engagement workshop observation/data).

##### Recruitment

Recruitment will occur through organisations, groups, social media, word of mouth and other channels. Some recruitment opportunities are also expected to emerge through the community contextual mapping process. This approach is intended to capture diverse perspectives, which enables the research team to understand the community/neighbourhood environment and available youth resources, including the barriers and facilitators that may impact access to and engagement with these resources.

For community engagement workshops and key informant interviews, flyers describing the study and participant eligibility criteria will be shared via social media and posted on community/faith-based organisations/institutions’ notice boards. The team will also perform in-person recruitment and direct mailing based on referrals. Flyers will provide information about the study and extend an invitation to contact the research team if interested in participating. Social media platforms such as Facebook and *X, formerly known as Twitter,* which host groups for school/service clubs, will also be used in the recruitment of participants. Once a participant indicates a willingness to participate, a research assistant will schedule an appointment to assess eligibility and obtain consent and subsequent workshop attendance. There is no planned monitoring of participant characteristics during the recruitment beyond the inclusion criteria stated.

##### Data collection

Data are collected via multiple approaches to ensure *synthesis* of rich descriptions and perspectives of youth and key stakeholders:

- *Community engagement workshop:* the workshop facilitator will present the workshop agenda and objectives. The facilitator will also use guided discussion questions based on the six dimensions of the community readiness model^35^: (1) community efforts; (2) community knowledge of the efforts; (3) leadership; (4) community climate; (5) community knowledge about the issue; and (6) resources related to the issue (online supplemental file 1). After each topic is introduced, participants will be placed in small discussion groups to brainstorm and list barriers and facilitators to SHAL for youth in their own community. A member of the research team (notetaker) will write all ideas on a communal flip chart as they are shared by participants. Participants will then identify their priority issues within each category by voting and placing a sticker next to their preferred options. The items identified as high priority will be identified in this manner.
- *Key informant interviews:* a semi-structured key informant interview guide will use aspects of the community readiness assessment and include questions designed to understand: (1) ease of access to fruits and vegetables, consumption of fruits and vegetables, sources (e.g., market vs grown), price; (2) UPF cost, perceptions of nutritional benefit, availability and food insecurity; (3) approaches to environmental sustainability, recycling and packaging; access to green space, tree canopy and the value of a home energy audit; (4) active transportation; and (5) leisure time physical activity and sedentary behaviours. We will also seek to understand how lifestyles change with season (e.g., winter in Canada and hurricane season in the Caribbean) (online supplemental files 2–4). Key-informant interviews will be used to supplement/augment information gathered from the community engagement workshops. This may be due to a group not being represented during the community engagement workshop due to logistic or scheduling challenges. Consequently, the final questions for the key informant interviews will be based on information gathered from the community engagement workshop.

##### Data analysis

Community engagement workshops and key informant interviews will be summarised using content and thematic analysis.^36 37^ Formative workshop data will be captured through the generation of priority lists and summary notes maintained by notetakers. Key informant interviews will be audio-recorded. Recorded interviews will be transcribed verbatim and open-coded by study staff using NVivo V.12 software (QSR International, Brisbane, Australia). Content and thematic analysis will be used for the community engagement workshop session summary notes and semi-structured key informant interviews.^36^ The overarching analytical process will be inductive. The team will read and code transcripts, sentence-by-sentence, to identify and examine codes as subthemes and themes.

##### Anticipated outcome

Identification of barriers and facilitators to SHAL and possible solutions based on the perspectives of youth and community stakeholders (i.e., community members, community leaders, organisation leaders, policy actors/makers).

### Stage 2—Co-design of the SCORE! Youth SHAL Intervention

This stage utilises a descriptive qualitative study to understand priorities, gaps and opportunities for SHAL as seen through the lenses of youth who are positioned to implement the SHAL intervention.

#### Population

Youth (15–25 years) currently residing in one of the selected neighbourhoods.

#### Sampling strategies and framework

The study will employ purposive and snowball sampling strategies. A purposive sampling strategy will be used to recruit youth residing in the study communities. This approach enables the research team to identify and invite participants who meet the inclusion criteria, including those referred by community partners, partner organisations, or members of the research team.^38^ The snowball sampling technique will be used as a secondary sampling technique to identify additional study participants through referrals by individuals who have agreed to participate. This sampling approach is useful where there might be difficulty in identifying participants and elicit ‘hidden voices’ in the population of interest. For workshops, a sample size of 10 participants per workshop per neighbourhood is the goal.

#### Recruitment

Potential participants will be recruited using the same methods described for the community engagement workshops. Youth who participated in the previous stage (community engagement) are also eligible to participate in the co-design workshops.

Once a participant is recruited into the study and provides assent/informed consent, a member of the research team communicates the scheduled workshop session for the participant. The co-design workshop will consist of a 60 min workshop with five segments: (1) summary of stage 1 findings along with introduction of initially planned SHAL intervention; (2) reflection on information shared and youth role in their community; (3) small group breakout session to discuss prioritized activities needed to support the implementation of the SCORE! Youth SHAL intervention; (4) large group session; and (5) summative/bringing it all together.

#### Data collection

We will use an inquiry-based learning approach that emphasises a youth-centred, solutions-focused discussion. At the beginning of each workshop, a trained facilitator presents the goals and expectations of the workshop. Based on information gathered from the community engagement stage, summary points will be presented in a manner suitable for the audience (e.g., video, live presentation, sharing circle, etc.) at the co-design workshop session for discussion and modification. Participants will be asked to reflect on and discuss the information shared. Additionally, participants will be presented with a menu of proposed interventions for the community (table 1). A research assistant or notetaker will document group responses.

**Table 1:** Proposed menu of interventions to present to co-design groups

	Food	Physical activity	Environmental sustainability impact
	*Reduced UPF use*	*Increased activity* *Reduced sedentary behaviours*	*Increased environmental stewardship*
Intervention	Education/awareness Community garden In-class/apartment garden Food skills Increased planning for hurricane and winter seasons	Education/awareness Walk or cycle to school/work Walking/hiking/cycling clubs Dance/play groups Fitness tracker Increased planning for hurricane and winter seasons	Education/awareness Circular strategies—reduce, reuse, recycle Increased planning for hurricane and winter seasons
Target behaviour	Reduce sugar-sweetened beverages Reduce ready-to-eat packaged foods Increase home-cooked meals	Active transportation to school/work Reduce screen time Increase physically active play Increase outdoor time/green space exposure	Reduce carbon footprint/greenhouse gas production Reduced use of single-use plastics and Styrofoam containers Increased use of locally grown, seasonal fruits/vegetables
Environmental sustainability impact	Circular strategies—reduce, reuse, recycle Compost	Reduced motor vehicle transport Reduced energy use	Circular strategies—reduce, reuse, recycle
Evaluation	Food Skills Questionnaire Knowledge, Attitudes and Behaviours Questionnaire Waste audit Change in home/school/community gardens Carbon footprint calculation Intergenerational learning	Physical Activity Questionnaire Knowledge, Attitudes and Behaviours Questionnaire Energy audit Carbon footprint calculation Intergenerational learning	Knowledge, Attitudes and Behaviours Questionnaire Energy audit Carbon footprint calculation Intergenerational learning

UPF, ultraprocessed foods.

Following the sharing and reflection period, participants will move into small groups to further explore the implementation of the SCORE! Youth SHAL intervention. Building on the touchpoints identified during the earlier discussion, each group will be asked to suggest ways to improve fellow youth experiences using the MoSCoW prioritisation (MoSCoW represents the following components: Must-Have, Should-Have, Could-Have and Won’t-Have).^39^ This approach has been used successfully for health care prioritisation.^39–42^

Each small discussion group will include a facilitator to guide the conversation and a notetaker to document participants’ views. Workshop participants will be invited to indicate their priorities within each MoSCoW category, and the results will be summarised using a tiered rating approach. In the final session, the workshop facilitator will present a synthesised summary to the larger group to clarify and confirm proposed strategies for enhancing the implementation of the SCORE! Youth SHAL intervention. At the conclusion of the workshop, all participants will complete a Post-Workshop Questionnaire (PPEET—Public and Patient Engagement Evaluation Tool, Participant Questionnaire).^43^ A secondary PPEET questionnaire (Project Questionnaire) will be completed by the research team, who served as workshop organisers.^43^ The PPEET tools assess the extent to which the intervention development process is transparent, collaborative and accountable to the community; requires a reasonable amount of time and resources and is perceived as offering a potential return on investment.^43^

#### Data analysis

Data from the co-design workshop will be analysed using NVivo V.12. Data will be coded according to the six steps for conducting thematic analysis as outlined by Braun and Clarke.^36^ Researchers will organise data into codes, and those codes into larger themes, which will connect with the study’s research questions. A codebook will be kept, maintaining the organisation of themes.

#### Evaluation of the codesign workshops

A summative analysis of responses to PPEET questionnaire items will be prepared. The PPEET Questionnaire synthesises quantitative scores and qualitative themes to provide an understanding of the workshop’s effectiveness in achieving the session objectives and the participants’ experience.

#### Output

Using our phase 1 findings, we will develop an intervention that promotes SHAL among youth. We will build on existing program of activities listed in ‘table 1 Proposed Menu of Interventions to Present to Co-design Groups’; see row labelled ‘intervention’: For nutrition, existing programs that teach healthy eating for youth, gardening skills and food skills will be presented to youth and adapted based on feedback. For physical activity, existing approaches to teaching ways to become more active and reduce sedentary behaviours and increase green space exposure will be presented to youth and modified for context. For environmental sustainability impact, lessons on sustainability, developed by The Starfish Canada, a pan-Canadian charity that has been working with schools since 2010, will form the base of the activities.^44^ Where appropriate, local organisations doing similar work will be consulted.^45^

#### Anticipated outcome

SCORE! Youth SHAL intervention co-designed with youth and for youth.

### Data management

Data collection and management will be done using SharePoint and REDCap (Research Electronic Data Capture). SharePoint is a system that assists with file organisation, controls user access and automatically tracks versions to preserve data integrity. In our study, it will be used to house qualitative data.

REDCap is a secure web application for building and managing online surveys and databases. The institution associated with the respective sites has a licence for REDCap software and has been using it in various small and large, cross-sectional and longitudinal studies. Using REDCap, data will be entered anonymously in the site-level portal by SCORE! Youth SHAL research personnel. All participants will be identified only by an identification number. Only the local principal investigator, coinvestigator and study manager/coordinator will have access to the database. The database will be designed with built-in data checks for real-time data quality assurance.

### Ethical considerations

The study has received ethical approval from McMaster University through HiREB (Project ID: 17774—8 November 2024); Brock University (Project ID 24–153–25 November 2024); The UWI, Mona Campus Research Ethics Committee (Project ID: CREC-MN.003,2024/2025—30 January 2025) and Ministry of Health and Wellness, Jamaica, Advisory Panel on Ethics and Medico-Legal Affairs (Project ID 2024/31—14 May 2025). The research team and study will adhere to the principles outlined in the Tri-Council Policy Statement: Ethical Conduct for Research Involving Humans. Additionally, study participants will be selected to participate only because of the specific purpose of the study.

## Discussion

This protocol paper outlines a novel, multimethod CBPR approach to addressing interconnected public health and environmental challenges faced by youth from low and middle-income countries (LMICs) and high-income countries (HICs). By focusing on reducing consumption of UPF, increasing physical activity and promoting environmental stewardship, the intervention targets multiple socially and structurally rooted behavioural risk factors for NCDs.

Implementing complex, equity-focused interventions in diverse, under-resourced settings requires careful attention to context, feasibility, and sustainability.^46 47^ This study addresses these concerns by embedding youth and youth service stakeholders/community members as co-designers and implementation partners, using a system-informed approach. Such participatory strategies have been shown to improve the contextual fit, acceptability and effectiveness of health interventions, particularly among equity-deserving populations.^48–50^ Additionally, the study focuses on a population (youth) at a critical juncture in the life cycle when health behaviour patterns are solidified and set the stage for lifelong health.^3^ Additionally, this study will generate insight into the development of chronic disease risk factors in this demographic. This is particularly important because, at this life stage, cumulative exposure to these risk factors remains relatively low, leaving ample opportunity to achieve long-term benefits through early intervention and primary prevention.

Our use of CBPR for phase 1 of the study (community engagement and co-design) and its extension to phase 2 (implementation) is guided by the RE-AIM framework (Reach, Effectiveness, Adoption, Implementation and Maintenance) and implementation outcomes such as feasibility, fidelity and acceptability. This approach reflects a growing shift towards adaptive, context-responsive implementation strategies that use community involvement and local needs.^46 47^ Additionally, this approach allows us to frame the intervention through a life-course and Global Syndemic lens, thereby broadening its relevance beyond individual-level behaviour change to address upstream social, economic and ecological determinants.^51^ This interdisciplinary framing also aligns with calls for integrated public health and planetary health solutions.

For the community engagement phase, all attributes of the community readiness model are utilised except for the overall score/or level of readiness (because it does not apply).^35^ The team has used suggested activities such as engagement of youth and community members/stakeholders through interviews (informal and formal), workshops, SHAL asset mapping and concepts centred around perception of one’s community based on SHAL component for youth to help guide workshop and key informant interview sessions.

Although the components of our intervention have been shown to work in other settings, evidence that these interventions in LMICs and among HICs remains limited.^52–57^ Consequently, community engagement activities should focus on understanding the contextual nuances of implementing a proven-effective intervention rather than on providing information for developing a new intervention.

Anticipated challenges include managing cross-sector partnerships, sustaining youth engagement and navigating institutional structures that may resist power-sharing or participatory methods. However, the study has established a youth advisory council that will operate alongside research activities. This council is expected to: (1) enhance recruitment and study reach; (2) foster trust within the community (its members live locally); (3) ensure continuity beyond the study duration (they function independently of the research team); and (4) enable iterative adaptation (reviewing and vetting research instruments and materials before their use).^58^ Ultimately, findings from this study are expected to inform the refinement and scale-up of integrated health–environment interventions and contribute to implementation science frameworks that explicitly centre equity, participation and system transformation.^59 60^ Youth represent a critical period for establishing lifelong health behaviours and preventing chronic disease. However, many health promotion initiatives do not adequately reflect the lived experiences of youth in LMICs or HICs. This study is unique in its use of community mapping, engagement workshops and codesign processes to develop culturally relevant and contextually appropriate SHAL interventions across diverse communities in Canada and Jamaica. By positioning youth as active partners throughout the research process, this study has strong potential to inform scalable, community-driven approaches that promote healthy active living, environmental sustainability and chronic disease prevention.

## Supplementary material

10.1136/bmjopen-2026-119261 online supplemental file 1

10.1136/bmjopen-2026-119261 online supplemental file 2

10.1136/bmjopen-2026-119261 online supplemental file 3

10.1136/bmjopen-2026-119261 online supplemental file 4
