# Supplementary material for: Developing a youth sustainability healthy active living intervention for chronic disease prevention and environmental sustainability through community engagement and codesign: protocol for a multimethod community-based participatory research study in Canada and Jamaica
Source: BMJ Open. 2026 Oct 7;16(10):e119261. doi: 10.1136/bmjopen-2026-119261 (PMC13646957; doi:10.1136/bmjopen-2026-119261)
Supplement: Supplementary file 1 [file bmjopen-16-10-s001.pdf]

Supplemental 1: SCORE Youth SHAL! Questions for Community Engagement Workshop

Youth in your community face a dual burden of chronic disease and effects of climate change. We are looking for ways to help engage youth in solutions to the climate change crisis that can be enacted locally, and ways to enhance sustainable healthy active living for youth.

COMMUNITY EFFORTS/KNOWLEDGE

1. Using a scale from 1-10, how much of a concern is this issue in your community (with 1
   being “not at all” and 10 being “a very great concern”)? Please explain
2. Please describe the efforts that are available in your community to address this issue.
3. How long have these efforts been going on in your community?
4. Using a scale from 1-10, how aware are people in your community of these efforts (with 1 being "no awareness" and 10 being "very aware")? Please explain.
5. What does the community know about these efforts or activities?
6. What are the strengths of these efforts?
7. What are the weaknesses of these efforts?
8. Who do these programs serve?
9. Would there be any segments of the community for which these efforts/services may appear inaccessible?
10. Is there a need to expand these efforts/services? If not, why not?
11. Is there any planning for efforts/services going on in your community surrounding this issue? If yes, please explain.
12. What formal or informal policies, practices and laws related to this issue are in place in your community, and for how long?
13. Are there segments of the community for which these policies, practices and laws may not apply?
14. Is there a need to expand these policies, practices and laws? If so, are there plans to expand them? Please explain.
15. How does the community view these policies, practices and laws?

LEADERSHIP

16. Who are the "leaders" for climate change and SHAL efforts for youths in your community?
17. Using a scale from 1 to 10, how much of a concern is this issue to the leadership in your community (with 1 being “not at all” and 10 being “of great concern”)? Please explain.

18. How are these leaders involved in efforts regarding this issue? Please explain. (For example: Are they involved in a committee, task force, etc.? How often do they meet?)
19. Would the leadership support additional efforts? Please explain.

<u>COMMUNITY CLIMATE</u>

20. Describe ________________________________ (name of community).
    a. I want you to take a moment and imagine your neighbourhood an example community for SHAL! What would this look like to you? What changes would it take to get you there?
21. Are there ever any circumstances in which members of your community might think that this issue should be tolerated? Please explain.
22. How does the community support the efforts to address this issue?
23. What are the primary obstacles to efforts addressing this issue in your community?
24. Based on the answers that you have provided so far, what do you think is the overall feeling among community members regarding this issue?

<u>KNOWLEDGE OF THE ISSUE</u>

25. How knowledgeable are community members about this issue? Please explain.
26. What type of information is available in your community regarding this issue?
27. What local data are available on this issue in your community?
    How do people obtain this information in your community?
28. What policy changes have you seen that have improved SHAL activities in your neighbourhood/ward/Parish?
29. Are you aware of any upcoming work relating to SHAL in your community?
30. Is there anything else you would like to share with us?
