# Supplementary material for: Developing a youth sustainability healthy active living intervention for chronic disease prevention and environmental sustainability through community engagement and codesign: protocol for a multimethod community-based participatory research study in Canada and Jamaica
Source: BMJ Open. 2026 Oct 7;16(10):e119261. doi: 10.1136/bmjopen-2026-119261 (PMC13646957; doi:10.1136/bmjopen-2026-119261)
Supplement: Supplementary file 2 [file bmjopen-16-10-s002.pdf]

Supplemental 2: SCORE! Youth SHAL Key Informant Interview Guide - Policymakers

1. What policy changes have you seen that has improved SHAL activities in your neighbourhood/ward/Parish?
2. What policy changes would you like to see implemented that have not been? What do you perceive to be the barriers to implementation?
3. What do you perceive to be “easy wins” from a policy perspective to support SHAL activities in youth?
4. What stakeholder groups/community partners are you engaged with/working with to advance SHAL in your community?
5. What is your office/program doing that has successfully engaged youth in the community/
6. Who are positive role models in this community, and have they done anything related to the call?
7. Where do youth in this neighbourhood hang out or socialize?
8. What are the most popular programs that youth participate in in your community?
9. Is there anything else you would like to share with us?
10. [Provide a brief description of SCORE Youth SHAL study] I want you to take a moment and imagine your neighbourhood an example community for SHAL! What would this look like to you? What changes would it take to get you there?
11. What aspects of SHAL do you think policy change would have the greatest impact? What’s standing in the way of that change?

Thank for taking the time to talk with me today.
