# Supplementary material for: Developing a youth sustainability healthy active living intervention for chronic disease prevention and environmental sustainability through community engagement and codesign: protocol for a multimethod community-based participatory research study in Canada and Jamaica
Source: BMJ Open. 2026 Oct 7;16(10):e119261. doi: 10.1136/bmjopen-2026-119261 (PMC13646957; doi:10.1136/bmjopen-2026-119261)
Supplement: Supplementary file 3 [file bmjopen-16-10-s003.pdf]

Supplemental 3: SCORE! Youth SHAL Key Informant Interview Guide - Community Partners/Stakeholders

1. Tell us about physical activities (e.g., sports, exercise) your organization offers for youth in the community.
2. Does your organization offer space for youth to engage in these activities? If so, what spaces?
3. How do people or youths in the community access your programs
4. Tell us about any activities in your organization is involved in that aim to make the environment better (e.g., park cleanups, weeding)
5. Is your organization involved in growing or providing food, or food skills for your community? How so?
6. Where do youth tend to hang out in the community/neighbourhood?
7. Are there any places that are known as not being problematic for youth? Examples may be safety, noise, lighting, not welcoming for youth? If so, Why?
8. Do you find that the types of activities programming your organization offers changes a lot from season-to-season?
9. Does your organization offer opportunities for youth to get involved with coaching and mentoring younger children?
10. Is there anything else you would like to share with us?

Thank for taking the time to talk with me today.
