# Supplementary material for: Developing a youth sustainability healthy active living intervention for chronic disease prevention and environmental sustainability through community engagement and codesign: protocol for a multimethod community-based participatory research study in Canada and Jamaica
Source: BMJ Open. 2026 Oct 7;16(10):e119261. doi: 10.1136/bmjopen-2026-119261 (PMC13646957; doi:10.1136/bmjopen-2026-119261)
Supplement: Supplementary file 4 [file bmjopen-16-10-s004.pdf]

Supplemental 4: SCORE! Youth SHAL Key Informant Interview Guide - Youths

1. Tell us about physical activities (e.g., sports, exercise) that you participate in in your community. Are there any activities you do not participate in? If so, why?
2. Tell us about the places you go to engage in these activities.
3. Where are your favourite places to hang out with your friends in the community/neighbourhood?
4. How safe do you feel walking alone in your neighbourhood during the day? At night?
5. Tell us about any activities in your community that aim to make the environment better (e.g., park cleanups, weeding)
6. Where do you usually go to buy food?
7. Is it easy to find what you think are healthy foods close to where you live?
8. Are there any places that you don't feel welcome? Why?
9. Do you find that the types of activities you participate in change a lot from season-to-season?
10. Are there opportunities for you and your friends to get involved with coaching and mentoring younger children?
11. Is there anything else you would like to share with us?

Thank for taking the time to talk with me today.
