# Supplementary material for: Developing a youth sustainability healthy active living intervention for chronic disease prevention and environmental sustainability through community engagement and codesign: protocol for a multimethod community-based participatory research study in Canada and Jamaica
Source: BMJ Open. 2026 Oct 7;16(10):e119261. doi: 10.1136/bmjopen-2026-119261 (PMC13646957; doi:10.1136/bmjopen-2026-119261)
Supplement: Supplementary file 5 [file bmjopen-2026-119261.reviewer_comments.pdf]

PEER REVIEW HISTORY

BMJ Open publishes all reviews undertaken for accepted manuscripts. Reviewers are asked to complete a checklist review form and are provided with free text boxes to elaborate on their assessment. These free text comments are reproduced below.

ARTICLE DETAILS

Title (Provisional)

Developing a youth Sustainability Healthy Active Living intervention for chronic disease prevention and environmental sustainability through community engagement and co-design: Protocol for a multi-method community-based participatory research study in Canada and Jamaica

Authors

Stennett, Rosain; Kandasamy, Sujane; Sherifali, Diana; de Souza, Russell J.; Desai, Dipika; Kwan, Matthew Y. W.; Smith, Joanne; Edwards, Thera; Gordon-Strachan, Georgiana; Bangdiwala, Shrikant; Wahi, Gita; Brook, Jeffrey; Sergeant, Myles; Krishanthan, Alisha; Desai, Sneha; Anderson, Simon G; Anand, Sonia S

VERSION 1 - REVIEW

**Reviewer**	**1**
**Name**	**Svobodova, Martina**
**Affiliation**	**Cardiff University, Centre for Trials Research**
**Date**	**10-Jun-2026**
**COI**	**None**

This is an interesting and worthwhile protocol. The participatory approach, youth involvement, and focus on both health and environmental sustainability are notable strenghts. The manuscript is well written and the overall study design appears appropriate to address the study aims.

I have a small number of suggestions:

1. The study objectives and intended outputs could be stated more explicitly. At different points the manuscript refers to understanding community perspectives, identifying barriers and facilitators, adapting intervention components, and co-designing an intervention. A clearer statement of the primary objectives or research questions would be helpful.

2. As the study is described as a co-design process, it would be useful to clarify which intervention components will be co-designed with participants and which are already established.

3. The authors could provide further detail on how recruitment/participant selection will ensure a broad range of perspectives from the populations intended to benefit from the intervention. Also please add whether participant characteristics will be monitored during recruitment and acknowledge the potential influence of intersectionality.

4. More detail on how the various qualitative and quantitative data sources will be integrated, and how findings from earlier stages will inform subsequent stages of the project, would strengthen the methods section.

5. The rationale for sample size could be reconsidered. Terms such as representativeness and generalisability may not be the most appropriate language for a qualitative participatory study, and a rationale based on diversity of perspectives and depth of engagement may be more suitable.

6. The manuscript would benefit from adding the study timeline, recruitment and anticipated completion dates.

VERSION 1 - AUTHOR RESPONSE

**Reviewer**	
This is an interesting and worthwhile protocol. The participatory approach, youth involvement, and focus on both health and environmental sustainability are notable strengths. The manuscript is well written and the overall study design appears appropriate to address the study aims.	N/A
I have a small number of suggestions:	N/A
1. The study objectives and intended outputs could be stated more explicitly. At different points the manuscript refers to understanding community perspectives, identifying barriers and facilitators, adapting intervention components, and co-designing an intervention. A clearer statement of the primary objectives or research questions would be helpful.	Thank you for your comment.<br><br>We have updated the last paragraph of the introduction to state the overall study aim and primary outcome:<br>"Study Aim: The aim of this project, SCORE! Youth SHAL (Phase 1), seeks to co-design with youths (ages 15 – 25 years) and stakeholders, an intervention program that increases knowledge and tools for action to increase opportunities for sustainable practices (i.e., environmental stewardship) and healthy active living (i.e., including production and consumption of local fruits and vegetables, decreasing UPF consumption, etc.). We will engage with the target communities through co-design to achieve this goal.<br>Primary Outcome: The SCORE! Youth SHAL intervention." pages 6 – 7

	Specific objectives/aims and anticipated outcome for phases: 1) Community Engagement and 2) Co-design have been incorporated throughout the manuscript.<br><br>Community engagement<br>"Specifically, this stage aims to: 1) understand the community-level contextual factors (i.e. assets) that impact SHAL; 2) identify strengths, levers, challenges, and barriers the communities face regarding SHAL; and 3) work with all relevant actors to identify levers and brainstorm approaches to implementation of the SCORE! Youth SHAL intervention." page 9<br><br>Added<br>"Anticipated Outcome:<br>Barriers and facilitators to SHAL and possible solutions based on the perspectives of youths and community stakeholders (i.e. community members, community leaders, organization leader, policy actors/ makers)." page 12<br><br>Co-design<br>"… to understand priorities, gaps and opportunities for SHAL as seen through the lenses of youths that are positioned to implement the SHAL intervention." pages 12 – 13<br><br>Added<br>"Anticipated Outcome: SCORE! Youth SHAL intervention co-designed with youth and for youth."
2. As the study is described as a co-design process, it would be useful to clarify which intervention components will be co-designed with participants and which are already established.	Thank you for your comment the Outcome section of the manuscript has been modified to state the following:<br><br>"Using our Phase 1 findings, we will consider several approaches to develop an intervention that promotes SHAL and reduces NCD risk factors in cities. We will build upon existing programs of activities listed in "Table 1 Proposed Menu of Interventions to Present to Co-design Groups" see row labelled "intervention":

	For nutrition, existing programs that teach healthy eating for youth, gardening skills, and food skills will be presented to youth and adapted based on feedback. For physical activity, existing approaches to teaching ways to become more active and reduce sedentary behaviours and increase green space exposure will be presented to youth and modified for context. For environmental sustainability impact, lessons on sustainability (reduce, reuse, recycle), developed by Starfish, a pan-Canada charity that has been working with schools for the past 12 years, will form the base of the activities (44). Where appropriate local organizations doing similar work will be consulted (45)." page 16
3. The authors could provide further detail on how recruitment/participant selection will ensure a broad range of perspectives from the populations intended to benefit from the intervention.	Thank you for this observation,<br><br>We have included the following:<br>"Multiple approaches will be taken to data collection (community engagement workshop and key-informant interviews) and participant sampling (convenience and/or purposive using varied organizations, groups, social media, word or mouth, etc. some of which is likely to get from the community contextual mapping). This approach is chosen to capture diverse perspectives that will help the research team to understand the community/ neighbourhood environment and youth resources through the barriers and facilitators identified." page 11<br><br>For the community engagement workshops we will aim to have a representation of each of the groups listed.<br>"These will include **youths aged 15–25 years**, **youth leaders** who are actively involved in community activities (such as service clubs, societies, or faith based groups), and **community members, including parents and other residents of the participating neighbourhoods**. We will also engage **community partners** who work with youth in their professional roles—such as educators, social workers, and members of the public health team—as well as organizational leaders who oversee youth

	focused programs or services, such as school administrators or faith leaders. In addition, policy actors from relevant areas of governance (e.g., local members of parliament, ministries of education or health, or public health officials) will be invited to contribute their perspectives." page 10<br><br>In the Data Collection - Key informant interviews section of the manuscript we have included the following:<br>" Key-informant interviews will be used supplement/ augment information gathered from the community engagement workshops. This maybe, to a group not being represented during the community engagement workshop. Consequently, the final questions for the key information interviews will be based on information gathered from the community engagement workshop." page 12<br><br>For the co-design workshop session there is no planned stratified sampling by age group, gender, etc. It is anticipated that the Sampling strategies and framework planned will garner diverse perspectives.<br><br>"The study will employ **purposive and snowball sampling strategies**. A purposive sampling strategy will be used to recruit youths residing in the study communities. This approach enables the research team to identify and invite participants who meet the inclusion criteria, including those referred by community partners, partner organizations, or members of the research team (38). The snowball sample technique will be used as a secondary sampling technique to identify additional study participants through referrals by individuals who have agreed to participate. **This sampling approach is useful where there might be difficulty in identifying participants and elicit 'hidden voices in the population of interest.** For our workshops, a sample size of 10 participants per workshop per neighbourhood is the goal." page 13

Also please add whether participant characteristics will be monitored during recruitment and acknowledge the potential influence of intersectionality.	We have added the following statement at the end of the 'Recruitment' section:<br>"There is no planned monitoring of participant characteristics during the recruitment beyond the inclusion criteria stated." page 11
4. More detail on how the various qualitative and quantitative data sources will be integrated, and how findings from earlier stages will inform subsequent stages of the project, would strengthen the methods section.	This study uses a multi-method design in which qualitative and quantitative data are collected, analyzed, and integrated across two sequential stages. Quantitative data from the EPOCH and PPEETT questionnaires will be used to characterize community resources and evaluate participants' experiences of the co-design process. Qualitative data from community engagement workshops, key informant interviews, and co-design workshops will provide an in-depth understanding of community contexts, as well as barriers and facilitators to Sustainability Healthy Active Living (SHAL).<br><br>Findings from Stage 1 (community mapping, engagement workshops, interviews, and EPOCH data) will be synthesized to identify community priorities, available resources, and contextual factors influencing SHAL. These findings will directly inform Stage 2 by guiding the content, structure, and adaptation of the co-design workshops and intervention components.<br><br>Data from Stage 2, including co-design workshop outputs and PPEETT evaluations, will then be integrated with Stage 1 findings to assess feasibility, contextual fit, and stakeholder perspectives. Integration will occur through triangulation and comparison of findings across data sources to develop a comprehensive understanding of how SHAL interventions can be tailored to diverse community settings.
5. The rationale for sample size could be reconsidered. Terms such as representativeness and generalisability may not be the most appropriate language for a qualitative	Thank you for your keen observation, we have modified the statement to:<br>"The following sample size and approach will allow for varied stakeholder

participatory study, and a rationale based on diversity of perspectives and depth of engagement may be more suitable.	representability, and will be sufficient to achieve data saturation…"
6. The manuscript would benefit from adding the study timeline, recruitment and anticipated completion dates.	The manuscript has been modified and to include:<br>"Study timeline: The study is expected to start recruitment September 2024 at least one site and end March – July 2026 depending on site." page 8
